# Missed opportunities in child healthcare

**DOI:** 10.4102/phcfm.v6i1.537

**Published:** 2014-08-05

**Authors:** Linda Jonker, Ethelwynn L. Stellenberg

**Affiliations:** 1 Faculty of Health Sciences, Department of Nursing, Stellenbosch University, South Africa

## Abstract

**Background:**

Various policies in health, such as Integrated Management of Childhood Illnesses, were introduced to enhance integrated service delivery in child healthcare. During clinical practice the researcher observed that integrated services may not be rendered.

**Objectives:**

This article describes the experiences of mothers that utilised comprehensive child health services in the Cape Metropolitan area of South Africa. Services included treatment for diseases; preventative interventions such as immunisation; and promotive interventions, such as improvement in nutrition and promotion of breastfeeding.

**Method:**

A qualitative, descriptive phenomenological approach was applied to explore the experiences and perceptions of mothers and/or carers utilising child healthcare services. Thirty percent of the clinics were selected purposively from the total population. A convenience purposive non-probability sampling method was applied to select 17 mothers who met the criteria and gave written consent. Interviews were conducted and recorded digitally using an interview guide. The data analysis was done using Tesch's eight step model.

**Results:**

Findings of the study indicated varied experiences. Not all mothers received information about the *Road to Health* book or card. According to the mothers, integrated child healthcare services were not practised. The consequences were missed opportunities in immunisation, provision of vitamin A, absence of growth monitoring, feeding assessment and provision of nutritional advice.

**Conclusion:**

There is a need for simple interventions such as oral rehydration, early recognition and treatment of diseases, immunisation, growth monitoring and appropriate nutrition advice. These services were not offered diligently. Such interventions could contribute to reducing the incidence of child morbidity and mortality.

## Abstrait

**Opportunités manquées dans le domaine des soins de santé aux enfants**.

**Contexte:**

Différentes politiques, telles que la Gestion intégrée des maladies enfantines, ont été mises en place pour améliorer la prestation de services intégrés dans le domaine des soins de santé aux enfants. Au cours de la pratique clinique le chercheur a remarqué que les services intégrés ne sont pas toujours assurés.

**Objectifs:**

Cet article décrit les expériences des mères qui ont utilisé les services de santé généralisés aux enfants dans la zone métropolitaine du Cap en Afrique du Sud. Ces services comprenaient le traitement des maladies; les interventions préventives telles que la vaccination; et les interventions incitatives, telles que l'amélioration de la nutrition et la promotion de l'allaitement.

**Méthode:**

Une approche phénoménologique, qualitative et descriptive a été appliquée pour étudier les expériences et perceptions des mères/personnes en charge qui utilisent les services de santé aux enfants. Trente pour cent des cliniques ont été sélectionnées à dessein parmi toute la population. On a appliqué une méthode pratique d’échantillonnage aléatoire et raisonnée pour sélectionner les 17 mères qui répondaient aux critères et ont donné leur consentement par écrit. On a effectué des entrevues et des enregistrements numériques à l'aide d'un guide d'entrevue. Pour l'analyse des données on a utilisé le modèle en huit étapes de Tesch.

**Résultat:**

Les résultats de l’étude ont montré des expériences variées. Les mères n'ont pas toutes reçu les renseignements sur le livret ou la carte « le Chemin de la Santé ». Selon les mères, il n'y avait pas de services de santé intégrés aux enfants. Les conséquences sont les opportunités manquées de vaccination, de fourniture de vitamines A, l'absence de contrôle de la croissance, d’évaluation de l'alimentation et de conseils nutritionnels.

**Conclusion:**

Il faut donc des interventions simples, comme la réhydratation orale, le dépistage précoce et le traitement des maladies, la vaccination, le contrôle de la croissance, et des conseils pour une nutrition appropriée. Ces services ne sont pas offerts assidûment. Ces interventions pourraient permettre de réduire le taux de morbidité et de mortalité chez les enfants.

## Introduction

Diligent provision of simple health interventions could reduce child mortality and morbidity. Examples of these interventions could include oral rehydration, early recognition and treatment of diseases, immunisation, growth monitoring and appropriate nutrition advice. However, despite improvements in healthcare delivery, implementation of appropriate programmes and having policies and legislation based upon best practices, South Africa has failed to reduce the maternal and under-five child mortality rate^1^. In fact, South Africa is one of 12 countries where the incidence of mortality in children younger than five years increased during the period 1990–2008.^2^ Missed opportunities may have contributed toward the incidence of mortality. However, important underlying conditions may be missed if the integrated approach to child healthcare is not practised.

### Setting

The research was conducted in the eastern side of the Cape Metropole in South Africa, which contains 10 clinics. The clinics included in the study are managed jointly by both the Provincial Government of the Western Cape and the Metro Municipal Health Services. Clinics from both authorities were selected in order to prevent prejudice and bias in the sampling process. The area was selected intentionally because children were referred to a hospital rendering primary-, secondary-and tertiary-level services. This provided the opportunity to obtain information about the child health services in the continuum of child healthcare services.

The researcher observed that policies and guidelines for rendering child health services may be disregarded in some clinical areas. It appeared as if integration of healthcare services was not practised in some places. Against this background, the researcher embarked upon a scientific study to determine if the observations made were a true reflection of services rendered.

### Key focus area

Mothers with children younger than two years of age and who utilised the healthcare offered at clinics, private general practitioners and hospitals were interviewed in order to obtain their actual experiences in the utilisation of these services.

### Contribution to the field

The findings of this research paper could create awareness for healthcare providers, policy makers in health and Nursing and Medical Education Institutions about the clients’ perception of services. By identifying experiences of those utilising the services, insight could be provided to practitioners and policy makers regarding the challenges faced by the users. Healthcare providers could use the information to reflect on their own practice measured against policy guidelines. Integrated comprehensive healthcare delivery could contribute toward minimising missed opportunities and, ultimately, toward helping to decrease morbidity and mortality in children younger than five years of age.

### Background

Many of the deaths in the under- five population have been avoidable and could be attributed to the incorrect implementation of health programmes.^3^ For example, separating the provision of curative and preventive paediatric care has resulted in many missed opportunities for immunisation.^4^ Fewer opportunities are missed if immunisation and nutrition services are available all day, rather than for limited periods of the week. Human Rights Watch cautions that the health system's inability to ensure accountability, oversight and effective response contributes to morbidity and mortality.^5^ Countries that made the most progress in reduction of child mortality were those that had higher coverage of integrated primary healthcare. The strongest predictor of changes in the under-five mortality rates were improvements in access to and provision of all the clinical services.

The healthcare of children is a parental responsibility, but society and the government have a moral responsibility to ensure that the rights of children according to the Constitution are protected. Child mortality is a basic reflection of the health status of children and an alternative marker of the quality of healthcare.^6^

Changes in society have influenced healthcare delivery to children. Prior to 1994, the plight of women and children was caused by a variety of social problems which included poverty, lack of nutrition and sanitation, unemployment, lack of immunisation programmes and unsafe water. Improvements after 1994 can (amongst others) be ascribed to the implementation of free services, the Expanded Programme on Immunization and Integrated Management of Childhood Illnesses programme.^7^ South Africa declared Primary Health Care (PHC) as the centrepiece of all its health policies since 1994.^8^ Key health promotion activities include: immunisation, the Integrated Management of Childhood Illness (IMCI) strategy, childhood infection prevention, neonatal health and developmental screening, growth monitoring and nutrition.^9^

The availability of programmes and policies does not imply that services are rendered as envisaged. An important way of evaluating the implementation of a programme is by obtaining detailed information on the clients’ experiences. Information on the experiences of the persons affected by the programme gives a more meaningful evaluation of what takes place during implementation.

Problems with the implementation of programmes within the healthcare package for children influence the quality of services rendered. Despite the fact that integrated PHC was adopted as a vehicle for service delivery, a lack of integration of services still exists. There is a need for high quality healthcare that will minimise the inequalities in society (wealth and social privileges) that impact on child health.^10^ Concerns regarding the rendering of child services could also be related to the quality of the data collected, which influences healthcare planning. The geographical access to services does not influence mortality but rather, the quality of care plays a significant role in the child mortality rates. This may be significant as there is a tendency to regard services in metropolitan areas as good, because of accessibility to primary healthcare.^11^

Policies direct or indicate the context and mode of implementation. Deviating from the intended methodology could result in unintended consequences. An example of this is where separating the provision of curative and preventive paediatric care could result in many missed opportunities for immunisation. Fewer opportunities are missed if immunisation and nutrition services are available all day, every day, rather than for a limited period each week.^4^ A survey in Ghana revealed that logistical challenges at hospitals, a shortage of nurses and a lack of knowledge were responsible for missed opportunities.^12^

Another aspect that may influence the implementation of policy directions could be monetary in nature. Sustainable funding for integrated programmes could be a problem, as funders tend to fund individual programmes. These programmes may then be implemented as a vertical service and related healthcare aspects may not be attended to.^13^ South Africa spends healthcare funds on quaternary (highly-specialised tertiary) and curative healthcare which should preferably be spent at a PHC level, where more people can be helped.^14^ Despite the fact that South Africa spends 8.7% of its Gross Domestic Product (GDP) on health, its healthcare outcomes are not as good as countries that are poorer and spend much less on health. Contributing factors are, amongst others, poor training of nurses and midwives; a curative rather than preventive approach; a lack of services providing education to schools and communities on nutrition; and a lack of infrastructure, equipment and consumables.^15^ Consequently, the integration of services may be compromised.

### Ethical considerations

The proposal was submitted to the Health Research Ethics Committee for Human Research at University of Stellenbosch for ethics clearance (N10/11/392), as well as the Metro Municipal Health Services (ID 10230) and the Provincial Department of Health of the Western Cape (RP 29/2011).

Informed consent was obtained to both conduct and make a digital recording of the interview. Respondents were provided with the ability to contact the researcher for feedback. Privacy and confidentiality, as well as limited access to research documents, were explained.

## Research methods and design

A qualitative, descriptive phenomenological approach was applied in order to explore the experiences and perceptions of mothers utilising child health services for children younger than two years. This design was appropriate as the researcher focused on actual services rendered. Phenomenologists conduct research in natural settings and consider it the best place to observe, ask questions and do interviews.^16^ In the experience of the researcher, service providers could rationalise omissions in adhering to guidelines to poor record keeping rather than poor compliance with policies.

A field diary was kept. Observations made by researcher were recorded and information obtained from the child's handheld *Road to Health* book.

For the purposes of this study and to improve understanding and ensure rigour in the study, the meaning of ‘mother’ is conceptualised as being the biological mother, adoptive mother, grandmother, foster mother, sibling or father who brings the child to the clinic (this terminology was adopted in order to incorporate the different caregivers who bring children for child healthcare services).

### Population and sampling

The target population was the mothers and/or carers of children younger than 24 months old who used the services at the clinics in a section of the eastern side of the metropolitan area in Cape Town, South Africa. A purposive sample of *n* = 3 (30%) of the clinics was selected from the total population. A convenience purposive non-probability sampling method was applied with regard to interviewing 17 mothers who met the criteria and gave written informed consent. According to De Vos et al.,^17^ a sample of *n* = 10 is adequate in qualitative research or until data saturation is met.

### Data collection

The researcher (an independent nurse practitioner) conducted and digitally recorded the interviews with each participant using an interview guide. In-depth, open-ended questions were developed by considering the literature, objectives, expert opinion and own experience in the field. Questions explored the experiences of the mothers about the services received. Observations and information from the child's *Road to Health* book were documented in the field diary. Participants’ responses were explored for interrelatedness during the course of the interview. An interview guide was used, as can be seen in Box 1.

*BOX 1:* Interview guide.

- Tell me about the *Road to Health* book of your child.
- Tell me for what services do you bring your child to the clinic.
- Tell me what happens when you bring your child to the clinic.
- Tell me what happens when you take your child to the doctor.
- Tell me what happens when you take your child to the hospital.
- Tell me what they told you about your child's weight.
- Tell me what they told you about your child's diet/food.
- Is there anything you want to share with me regarding the services at the clinics?
- Is there any suggestion you want to share regarding how the services can be improved?

### Data analysis

The data analysis was done using Tesch's eight step model,^18^ supported by the guidelines supplied by Burns and Grove.^19^ The analysis involved the transcription of digitally-recorded interviews according to the coding of the data, the generating of themes and subthemes, interpretation and organisation of data and the drawing of conclusions.

### Ensuring quality

A pre-test was conducted at one clinic, where two respondents were interviewed. The value of the pre-test was that it elicited shortfalls in the methodology and ensured rigour in the process.

Dependability was ensured by the careful description of each step of the data collection and analyses processes. The researcher completed the data analysis and coding processes. A colleague assisted by doing a similar process of assigning codes to the transcribed data. An expert in qualitative research and nursing reviewed the data analyses and the data coding processes. The expert evaluated the dependability of data analysis.

Conformability was maintained by keeping field notes during the interviews and data analysis processes; any preconceived ideas and observations were noted and bracketed. The Modelling Role-Modelling Nursing theory of Erickson, Tomlin and Swain, as mentioned by George's work on nursing theories,^26^ was utilised as the conceptual theoretical framework to facilitate its application to the broad population. Detailed records of the coding and analysis process were kept. The reflective memos, marginal remarks and the record of the data trail will assist if the processes should be replicated.

## Results

Interviews were conducted at three clinics. At clinic A, six participants were interviewed, four at clinic B and seven at clinic C, making it a total of 17 participants interviewed. Findings relate to participant characteristics and services utilised and their experiences at service delivery points.

### Participant characteristics and services utilised

Sixteen children were accompanied by their biological mother and one child by the grandmother. Eight mothers had only one child; eight had two children and one had five children. The age of the children ranged between three weeks and 18 months. The group appeared to consist of mothers who normally visited clinics and could provide information on their experiences.

All mothers used the clinic as a service provider. Eleven mothers also used private medical practitioners and eight mothers used the hospitals for the healthcare of their children. The hospital was used by choice and also after hours, if no clinic was open.

The mothers were interviewed about services received at all service providers as they form part of the continuum of care.

### Experiences of participants at service delivery points

The participants’ experiences were highlighted and discussed according to the classification of Binkin et al.,^6^ who found in a meta-analysis of studies that the interventions by health workers to reduce the under-five mortality rate can be grouped into three broad groups, namely: treatment for diseases; preventive interventions, such as immunisation; and promotive interventions, such as improvement in nutrition and promotion of breastfeeding. The missed opportunities could then be highlighted.

### Treatment for diseases

*Children with life-threatening conditions* require immediate action from mothers, as well as their healthcare providers. Various methods of educating mothers can be utilised, such as posters, pamphlets, peer education and health information messages in the *Road to Health* book. The mothers knew about these conditions and knew not to wait in queues if the child is very sick:

‘You tell them and after 10 minutes they say you must come this side – five minutes. So if very sick they help you quickly...’ (Participant B1, Female)

The information was depicted on the walls of the facilities and photographs assisted mothers in the recognition of sick children. Only one facility had no rehydration corner and the participants verbalised their confusion in this regard:

‘I sat as normal I went to the place indicated, place was locked. Said someone will see to you. I left after 90 minutes.’ (Participant A6, Female)

*Underlying illnesses* should be managed according to the IMCI protocol that requires that growth, nutrition and immunisations should be assessed at each visit. The findings reflected that mothers could not recall if integrated or holistic management was rendered when the child was ill. Missed opportunities regarding the dispensing of vitamin A indicated that an integrated service was not practised:

‘Look at everything … Do not discuss weight and feeds. Will ask if child is still on the breast.’ (Participant A1, Female)

### Preventive interventions

*Immunisation services* should be offered every day at all facilities. The mothers’ experiences with immunisation services indicate that the concept of ‘seamless health services’ does not exist as far as immunisation was concerned. One of the mothers at clinic C related that this was the second time that the child was not immunised as a result of staffing problems. Immunisation services were not offered at the hospitals and private practitioners:

‘Take child to doctor if in hurry. Clinic takes too long. Mostly doctors do not weigh or check immunisation. Examine and finish. Do not look at Road to Health card.’ (Participant A4, Female)

Mothers explained that the card might be checked, but they were then referred back to the clinics. The successes of the Expanded Programme on Immunization were seen in obtaining polio-free status; reduction in incidence of measles morbidity and mortality; and the decrease in neonatal tetanus incidence.^3^ The measles outbreak of 2009–2010 in South Africa is a direct consequence of low herd cover. The higher the immunisation coverage, the better the herd immunity appears to be.^20^ It was also found that children were not immunised because of the unavailability of vaccines:

‘…[*s*]upposed to come for her injections last week but they did not have any in stock.’ (Participant A5, Female)

The guidelines are clear on the level of child care to be offered. Healthcare guidelines and policies such as the Expanded Programme on Immunization (South Africa) require immunisation services to be available at all service delivery points such as clinics, community health centres and hospitals. The Department of Health supports the participation of the private sector.^21^ Information available on 10 November 2011 on the website of the Department of Health actually motivates parents to visit their clinic or medical practitioner to obtain immunisations. The public is assured that the nursing sister will take the child's weight, length and head circumference in order to determine if the child is growing at the expected rate.^22^

*Vitamin A and deworming* should be offered at six-monthly intervals. In this study it appeared as if the provision of vitamin A was overlooked as only one child received all the required dosages. In a survey on missed opportunities in a Cape Town clinic, it was found that separating the provision of curative and preventive paediatric care resulted in many missed opportunities for immunisation. One of the findings is applicable to the provision of vitamin A.^4^ The recommendations of this study appear not to have been implemented as integrated services were not rendered at some facilities, according to the lived experiences of the mothers. Nurses attend to queues, not to children, according to one participant:

‘… [*T*]hey have to do all the queues …’ (Participant B2, Female)

### Health promotion activities

The *Road to Health* book should be provided at the birth of the child. Postnatal care of the participants took place at private and public healthcare facilities. In public facilities, children were born at primary, secondary and tertiary levels of care. The mothers’ experiences indicated that information on the *Road to Health* book or card was not offered to every mother:

‘No, I asked, but they said it is my child's card...’ (Participant C3, Female)

‘… [*N*]othing, only said it is the clinic card. It must be used when I take the child to the clinic …’ (Participant C5, Female)

Mothers should receive information on aspects that will enable them to care for their children. The *Road to Health* book or card is an excellent source of information for mothers. However, it was found that this opportunity could be better utilised. The omission to explain the card could imply that mothers were not enabled to assess the health of the child or to practise preventive healthcare in order to improve their child's health and prevent unnecessary healthcare costs.^23^ Health information messages in the book provide valuable information about feeding, oral rehydration, play and stimulation and safety of the child. There are drawings of children with danger signs that prompt the mother when to take the child to a healthcare service provider:

‘Everything about her is in it; also messages if she is sick that I can read; her birth weight and stuff. Everything is in it …’ (Participant A3, Female)

*Nutrition advice* should be offered diligently. Weight and growth of children younger than 24 months should be assessed at each contact session, at least once a month. Mothers received information about the weight gain of the child, but feeding assessments and nutritional advice were not offered:

‘They did explain that every time I come to the clinic the nurse will weigh the baby and the baby must pick up weight and if it goes down it is bad...’ (Participant A5, Female)

‘No, I was actually planning to ask them if she had picked up …’ (Participant B4, Female)

The mothers revealed incorrect feeding practices but received no advice regarding how to correct the practices. Grandmothers and family members seem to be a frequent source or provider of nutrition information:

‘My mother told me to give him water …’ (Participant C7, Female)

Inadequate knowledge about appropriate foods and feeding practices are often a greater determinant of malnutrition than actual lack of food.^24^ Participants related in this study that although they had access to the services, the nutrition services were not provided. Therefore, the concern was that the mothers were not able to improve the nutrition status of the child as a result of the possible lack of knowledge.

*Monitoring growth* requires assessment of weight, length and head circumference. All children were weighed. No mother verbalised that the child's length or head circumference were measured. Mothers knew that the weight was plotted on the growth card, but the curve was not discussed:

‘Your child is growing well mommy [*mimicking the staff*].’ (Participant C1, Female)

Some mothers received no information about the weight gain. Three mothers had children with low weight for their age. The mothers were informed about the concern when the child's growth curved crossed the -2SD line:

‘Last time they said she is low weight for her age and then they discussed it …’ (Participant B3, Female)

In this specific instance, the curve indicated unsatisfactory weight gain in the preceding months, but no feeding assessments were done during that period. Numerous gaps existed in the implementation of growth monitoring and nutrition promotion within the South African context. These gaps included inaccurate assessment of the weight of the children, a failure to plot weights, the inability of nursing staff to have identified the nutritionally at risk, poor utilisation of the *Road to Health* card and poor communication with caregivers.^1^ The national guidelines indicate that growth monitoring is an integral part of the immunisation visit. Another aspect of growth monitoring includes health advice to mothers regarding good nutrition.^6^

## Discussion

### Missed opportunities

Table 1 summarises the missed opportunities found at the time of the study.

**TABLE 1:** Missed opportunities.

Child health service	Missed opportunities
Immunisations	12 of 17 child immunisations were not done as required for the child's age
Vitamin A provision	3 of 4 children did not received Vitamin A according to age
Information provided about *Road to Health* book	13 of 17 mothers received no information about the *Road to Health* book
Developmental assessments done	16 of 17 children had no developmental assessments done for the required age
Nutrition information and feeding assessment	7 of 17 could not recall any information given or feeding assessments done

South Africa adopted good policies and guidelines based upon best practices after 1994. Delivering healthcare is regulated by these policies and guidelines. These standards of provision can be confirmed as the minimum desired or acceptable level of healthcare. Two aspects must be taken into account when this is interpreted: firstly, the national norms and standards define what should be offered; and secondly, the local norms and standards define what is offered. This revolves around staff competency.^25^ Health managers are responsible to ensure that healthcare workers are competent to deliver healthcare according to minimum standards. The findings of the study indicated that services were not integrated. Applying guidelines to practised integrated services could have prevented these missed opportunities and could also have contributed to lower the morbidity and mortality rates in children. Countries that had made the most progress, as measured by the average annual reduction of mortality, were those which had higher coverage of integrated primary healthcare. They also found that the strongest predictor of changes in the under-five mortality rates were improvements in access to and provision of all the clinical services.^6^

### Recommendations

Rendering of child services should be done using an integrated approach. This approach is not entrenched and urgent attention is required to ensure adherence to policies. Every contact visit should be utilised for the checking of growth, nutrition and immunisation status.

Maternal services should be strengthened so as to ensure that health promotion and education activities are offered. This should include explaining the *Road to Health* book to the mother and/or carer.

The private sector, medical officers and hospitals should participate and render child services according to policy objectives as determined for child services. This should be supported by health managers who are responsible for ensuring that quality integrated healthcare is rendered to all children who utilise child health services. This approach is endorsed by the National Department of Health.^22^

### Limitations of the study

This research was limited to clinics in the eastern section of the Cape Metropole of the Western Cape Province, South Africa.

## Conclusion

Research was conducted in the eastern side of the Cape Metropole in South Africa. Mothers were interviewed in order to determine their experiences regarding the healthcare offered for their children younger than two years old. Despite improvements in healthcare delivery, implementation of appropriate programmes and having policies and legislation based upon best practices, South Africa has failed to reduce the maternal and under-five child mortality rate.^1^ Diligent provision of simple health interventions could contribute to a reduction in child mortality and morbidity.

Mothers’ experiences revealed that integrated comprehensive services were not practised. Healthcare workers tend to ascribe omissions in service delivery to poor record keeping. However, mothers’ experiences suggested that the services not rendered were missed opportunities that could be the result of not applying integrated services.

Each contact visit is an opportunity to ensure that all required services according to age are rendered and omissions are rectified. Clinics can be busy places, with long queues of people waiting for healthcare services. Mothers have to care for families and may find it difficult to spend long hours in queues.

Further research to determine why hospitals, obstetrical services and the private sector do not follow national policies is needed. The underlying reasons why service providers do not seem to adhere to policies should be determined and addressed. Identifying the root causes could assist in addressing the shortfalls in service delivery.

The provision of equipment and manpower at clinics may be a contributing factor and should be investigated.

Providing only one aspect of child healthcare may lead to missed opportunities with regard to providing immunisations, as well as failure to dispense important disease prevention drugs such as vitamin A and deworming tablets. The child's nutritional status and diet may not be assessed and the mother may not receive advice on appropriate nutrition according to the child's age. These missed opportunities contribute to the high incidence in child mortality and morbidity. South African healthcare policies require that integrated healthcare be rendered for children.
